# Association Study between Mucin 4 (*MUC4*) Polymorphisms and Idiopathic Recurrent Pregnancy Loss in a Korean Population

**DOI:** 10.3390/genes13060937

**Published:** 2022-05-24

**Authors:** Ji-Hyang Kim, Han-Sung Park, Jeong-Yong Lee, Eun-Ju Ko, Young-Ran Kim, Hee-Young Cho, Woo-Sik Lee, Eun-Hee Ahn, Nam-Keun Kim

**Affiliations:** 1 Department of Obstetrics and Gynecology, CHA Bundang Medical Center, CHA University, Seongnam 13496, Korea; bin0902@chamc.co.kr (J.-H.K.); happyiran@cha.ac.kr (Y.-R.K.); 2 Department of Biomedical Science, College of Life Science, CHA University, Seongnam 13488, Korea; hahnsung@naver.com (H.-S.P.); smilee3625@naver.com (J.-Y.L.); ejko05@naver.com (E.-J.K.); 3 Department of Obstetrics and Gynecology, CHA Gangnam Medical Center, CHA University, Seoul 06135, Korea; hycho.md@cha.ac.kr; 4 Fertility Center of CHA Gangnam Medical Center, CHA University, Seoul 06135, Korea; wooslee@cha.ac.kr

**Keywords:** *MUC4*, recurrent pregnancy loss, polymorphisms

## Abstract

Recurrent pregnancy loss (RPL) is the loss of two or more consecutive pregnancies before 20 weeks of gestational age. Our study investigated whether mucin 4 (*MUC4*) polymorphisms are associated with RPL. *MUC* polymorphisms (rs882605 C>A, rs1104760 A>G, rs2688513 A>G, rs2258447 C>T, and rs2291652 A>G) were genotyped in 374 women with RPL and 239 controls of Korean ethnicity using polymerase chain reaction-restriction fragment length polymorphism analysis and the TaqMan probe SNP genotyping assay. Differences in genotype frequencies between cases of RPL and the controls were compared. *MUC4* rs882605 C>A and rs1104760 A>G polymorphisms were associated with increased incidence of RPL in three and four or more pregnancy loss patients. The haplotype analyses showed a tendency for the allelic effect including the association of *MUC4* rs882605 A and rs1104760 G alleles with increased incidence of RPL. In addition, the *MUC4* rs882605 CA/*MUC4* rs2258447 CC genotype combination was associated with increased RPL prevalence. The two exonic polymorphisms lead to amino acid changes of protein and may act as pathogenic variants for RPL. In conclusion, the *MUC4* rs882605 C>A and *MUC4* rs1104760 A>G polymorphisms were associated with the susceptibility of RPL and we considered them as potential biomarkers for RPL.

## 1. Introduction

Pregnancy loss, also known as miscarriage or spontaneous abortion, is the loss of pregnancy before 20 weeks of gestational age [1]. Recurrent pregnancy loss (RPL) refers to two or more consecutive pregnancy losses [2]. The incidence of early pregnancy loss within 12 weeks of gestation is approximately 10–12% of pregnant women, and RPL occurs in 1% of pregnant women [3,4]. Etiological factors of RPL include advanced maternal age, immunological problems, endocrine dysfunction, and anomalies of maternal anatomy, chromosomes, and placenta [5,6,7]. Moreover, environmental factors such as infection, smoking, alcohol, psychological trauma, and stress may contribute to RPL [8,9,10]. However, the cause of RPL is unclear in 30–40% of cases, and recent studies suggest that genetic factors play an important role in the pathogenesis of idiopathic RPL [11,12].

Mucins are high-molecular-weight glycoproteins that lubricate the epithelial surfaces of the respiratory, gastrointestinal, and reproductive tracts [13]. Mucins are secreted by the epithelium cells of reproductive tissues, generating the mucus of the cervix and endometrium, which play important roles in reproductive physiology. Moreover, mucins act as inflammatory and immune response mediators and transcriptional and posttranscriptional regulators [14,15,16]. Therefore, abnormal mucus secretion may contribute to female infertility.

Mucin 4 (MUC4) is the major mucin present in the endometrial epithelium of multiple species with MUC1 and MUC16 [17,18]. Composed of 25 exons, *MUC4* is located in the q29 region of chromosome 3 and encodes a 930-kDa transmembrane glycoprotein [19,20]. The extracellular domain of MUC4 can interact with human epidermal growth factor receptor 2 (HER2) on the cell surface and modulate downstream cell growth signaling by and enhancing the activity of cell growth receptor complexes [21,22]. Cancer studies show that MUC4 is aberrantly expressed in various malignancies and validate MUC4 as a novel target for cancer diagnosis [21,23]. Because the extracellular domain of MUC4 is critical for HER2 interaction and cell invasiveness, defined single nucleotide polymorphisms (SNPs) in putative functional domains of MUC4 may play important roles in RPL.

In this study, we investigated the association between RPL and five *MUC4* polymorphisms (rs882605 C>A, rs1104760 A>G, rs2688513 A>G, rs2258447 C>T, and rs2291652 A>G) in Korean women. The five polymorphisms are located within the exons of *MUC4* and have more than 20% of minor allele frequency in the Korean population (Figure S1). Three *MUC4* polymorphisms (rs882605 C>A, rs1104760 A>G, and rs2688513 A>G), located on the second exon, were missense variants. The rs882605 C>A and rs2688513 A>G polymorphisms change the first base of the codon, converting valine (GTT) to phenylalanine (TTT) and serine (TCA) to proline (CCA), respectively. The rs1104760 A>G polymorphism changes the second base of the codon, converting isoleucine (ATC) to threonine (ACC). In contrast, both polymorphisms in the 20th (rs2258447 C>T) and 23rd (rs2291652 A>G) exons were synonymous variants that caused no amino acid changes.

## 2. Results

The baseline characteristics and clinical profiles of the RPL patients and controls are summarized in Table 1. The mean ages of the RPL patients and control populations were 33.21 ± 4.60 and 33.21 ± 5.44 years, respectively. Body mass index (BMI), number of previous pregnancy losses, concentrations of homocysteine, folate, total cholesterol, uric acid, and plasminogen activator inhibitor-1 (PAI-1), platelet count (PLT), prothrombin time (PT), and activated partial thromboplastin time (aPTT) are summarized in Table 1.

We investigated the genotype frequencies according to the number of pregnancy losses in the RPL patients (Table 2). In the analysis of the total RPL patients and controls, the five polymorphisms did not show a significant association with RPL risk. However, the *MUC4* polymorphisms rs882605 C>A [CC vs. CA: adjusted odds ratio (AOR), 1.746; 95% confidence interval (CI), 1.106–2.757; *p* = 0.017 and dominant model: AOR, 1.804; 95% CI, 1.159–2.809; *p* = 0.009) and rs1104760 A>G (AA vs. AG: AOR, 2.001; 95% CI, 1.281–3.125; *p* = 0.002 and dominant model: AOR, 1.880; 95% CI, 1.216–2.905; *p* = 0.005] were significantly associated with increased susceptibility to RPL in patients with three or more pregnancy losses. Moreover, the increased susceptibilities with rs882605 C>A (CC vs. CA: AOR, 1.887; 95% CI, 1.081–3.293; *p* = 0.026 and dominant model: AOR, 1.862; 95% CI, 1.080–3.210; *p* = 0.025) and rs1104760 A>G (AA vs. AG: AOR, 1.843; 95% CI, 1.062–3.196; *p* = 0.030 and dominant model: AOR, 1.757; 95% CI, 1.026–3.008; *p* = 0.040) were maintained in patients with four or more pregnancy losses.

**Figure 1:**
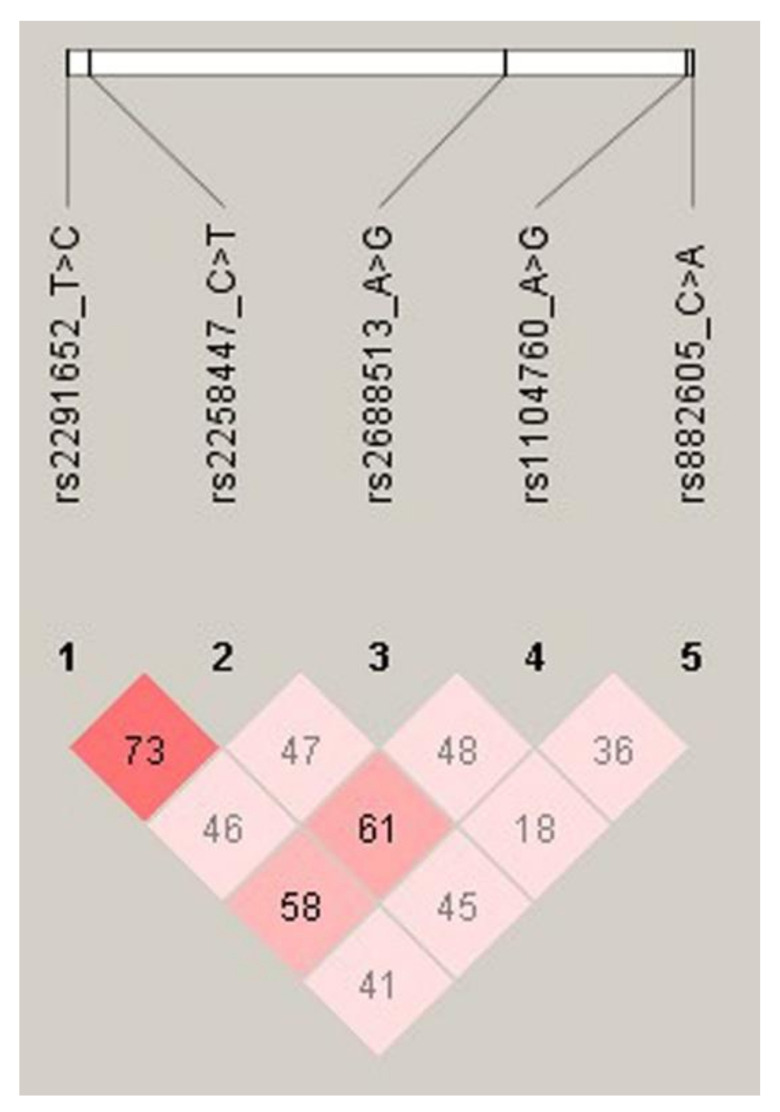
The linkage disequilibrium (LD) block structure consisted of the five SNPs located in *MUC4*.

We conducted haplotype analyses of the RPL patients and control subjects (Figure 1, Table 3 and Table S1). We found that haplotypes with increased odds ratio (OR) included the C-G and A-A of the rs882605 C>A and rs1104760 A>G polymorphisms. In the two alleles’ haplotype (rs882605 C>A/rs1104760 A>G), the C-G (OR, 1.628; 95% CI, 1.092–2.428; *p* = 0.016) and A-A (OR, 1.887; 95% CI, 1.222–2.914; *p* = 0.004) showed significantly increased RPL risk. In the three alleles’ haplotype (rs882605 C>A/rs1104760 A>G/rs2258447 C>T), the C-G-T (OR, 5.189; 95% CI, 2.448–11.000; *p* < 0.0001), A-A-C (OR, 5.394; 95% CI, 2.657–10.950; *p* < 0.0001), and A-G-C (OR, 2.990; 95% CI, 0.997–8.966; *p* = 0.040) were associated with increased RPL risk. In the four alleles’ haplotype (rs882605 C>A/rs1104760 A>G/rs2688513 A>G/rs2258447 C>T), the C-G-A-T (OR, 4.672; 95% CI, 1.381–15.800; *p* = 0.005), C-G-G-T (OR, 5.072; 95% CI, 1.975–13.030; *p* = 0.0002), A-A-A-C (OR, 6.578; 95% CI, 2.984–14.500; *p* < 0.0001), and A-G-G-C (OR, 15.360; 95% CI, 0.901–261.700; *p* = 0.004) showed significantly increased RPL susceptibility.

We conducted a genotype combination analysis of the RPL patients and the control subjects (Table 4). In the genotype combination analysis of rs882605 C>A and rs1104760 A>G, the CC/AG (OR, 1.878; 95% CI, 1.150–3.067; *p* = 0.012), CA/AA (OR, 1.890; 95% CI, 1.104–3.235; *p* = 0.020), and CA/GG (OR, 0.195; 95% CI, 0.041–0.936; *p* = 0.041) combinations were significantly associated with RPL risk. In the genotype combination analysis of rs882605 C>A and rs2258447 C>T, CC/CT (OR, 2.944; 95% CI, 1.592–5.444; *p* = 0.001) and CA/CC (OR, 5.441; 95% CI, 2.609–1.350; *p* < 0.0001) showed significantly increased OR. In the genotype combination analysis of rs882605 C>A and rs2291652 A>G, CC/AG (OR, 1.583; 95% CI, 1.026–2.441; *p* = 0.038) and CA/AA (OR, 2.505; 95% CI, 1.333–4.708; *p* = 0.004) were associated with increased RPL risk. In the genotype combination analysis of rs1104760 A>G and rs2688513 A>G, AA/AG (OR, 0.532; 95% CI, 0.297–0.953; *p* = 0.034) and AG/AG (OR, 2.157; 95% CI, 1.252–3.719; *p* = 0.006) were associated with RPL risk. In the genotype combination analysis of rs1104760 A>G and rs2258447 C>T, AG/CT (OR, 1.627; 95% CI, 1.031–2.570; *p* = 0.037) was associated with increased RPL risk.

We conducted an analysis of the difference levels of clinical factors according to the genotype of the *MUC4* polymorphisms. The rs2258447 C>T polymorphism was associated with gradually decreased aPTT levels according to the altered genotypes of rs2258447 (Table 5).

## 3. Discussion

In this study, we investigated the association between five *MUC4* polymorphisms (rs882605 C>A, rs1104760 A>G, rs2688513 A>G, rs2258447 C>T, and rs2291652 A>G) and RPL susceptibility in Korean women.

*MUC4* rs882605 C>A and *MUC4* rs1104760 A>G polymorphisms were associated with a high incidence of RPL in three and four or more pregnancy loss patients. The haplotype analyses showed a tendency for an allelic effect and that the *MUC4* rs882605 A and *MUC4* rs1104760 G alleles were associated with a high incidence of RPL. Similarly, the *MUC4* rs882605 CA/*MUC4* rs1104760 AA genotype combination was associated with increased RPL prevalence.

Mucin proteins are high-molecular weight epithelial glycoproteins with a high content of clustered oligosaccharides O-glycosidically linked to tandem repeat peptides rich in threonine, serine, and proline [24]. They are developmentally regulated and highly immunogenic [25,26,27]. Mucins are secreted by the epithelium cells of reproductive tissues, generating the mucus of the cervix and endometrium, which play important roles in reproductive physiology. Our data suggest an association between *MUC4* polymorphisms and RPL. Recently, several studies have shown that MUC4 can promote cell migration, change the endometrial environment, and create weak points in the epithelium, thus facilitating the failure of embryo implantation [18,28]. In addition, *MUC4* is associated with advanced stages of endometriosis [29].

Interestingly, the two polymorphisms rs882605 C>A and rs1104760 A>G, which are strongly associated with increased RPL risk (Table 2), are missense variants in the second exon of *MUC4*. Moreover, only the two polymorphisms are located on the first and second position of the codon, which change valine (GTT) to phenylalanine (TTT) and isoleucine (ATC) to threonine (ACC), respectively. Several studies have reported that two amino acid changes alter functionality and disease association in other proteins [30,31,32,33]. The two polymorphisms were found in two different long-loop regions in predicted secondary structures [29]. Therefore, the two *MUC4* polymorphisms may act as pathogenic variants through the structural changes of the MUC4 protein.

This study had several potential limitations. First, how the polymorphisms in *MUC* affect RPL development is still unclear, and a functional study of the SNPs to elucidate the pathogenesis of RPL was not conducted. Second, the study does not include information regarding the placental pathology, immunologic profiles, or *MUC* expression. Third, the population of this study was restricted to patients of the Korean population. Although the present study can contribute to assessments of the individual risk of RPL, further epidemiologic studies should be performed to confirm and expand our results. The role of MUC in the pathogenesis of RPL must be further investigated.

In conclusion, we identified genetic associations of *MUC4* rs882605 C>A and *MUC4* rs1104760 A>G polymorphisms with RPL prevalence in Korean women. Moreover, haplotype analysis showed that the *MUC4* alleles were significantly different between the RPL patients and controls. In addition, the *MUC4* combined genotypes showed a significant association in RPL.

## 4. Materials and Methods

### 4.1. Study Population

Genomic DNA was extracted from anti-coagulated peripheral blood using the G-DEX Blood Extraction Kit (Intron, Seongnam, South Korea). Polymorphisms were determined by polymerase chain reaction–restriction fragment length polymorphism (PCR–RFLP) analysis and the TaqMan probe SNP Genotyping Assay Kit (Applied Biosystems, Foster City, CA, USA) using the isolated genomic DNA as a template. The PCR primers were designed to amplify each SNP as follows: *MUC4* rs882605 C>A, forward 5′—CAC CTC AGC AGC CTT AGT AAT A—3′ and reverse 5′—TGA TGT TGT AAC CGG TGT G—3′; rs1104760 A>G, forward 5′—CCA CTT ACA GAT AGT GAT GTC TCC—3′ and reverse 5′—CTC AAA TCA ACA CCC TCA ACA C—3′; rs2688513 A>G, forward 5’—ACA TAA AGG CGA GGC AGT TG—3’ and reverse 5’—ACC CCT CTT CCT GTC ACC A—3’; rs2291652 A>G, forward 5’—GAA GCC CCA TCC AAC ACT GG—3’ and reverse 5’—GAC TCA CGG GCT GTC ACA TC—3’. All conditions for PCR amplification were based on initial denaturation at 95 °C for 15 min, followed by 35 cycles with denaturation at 95 °C for 30 s, annealing for 30 s, extension at 72 °C for 30 s, and a final extension at 72 °C for 5 min. The annealing temperatures for *MUC4* rs882605 C>A, *MUC4* rs1104760 A>G, *MUC4* rs2688513 A>G, and *MUC4* rs2258447 A>G were 58 °C, 55 °C, 54 °C, and 55 °C, respectively. The PCR products for rs882605, rs1104760, rs2688513, and rs2291652 were digested using the *Ssp*I, *Bcc*I, *Bst*NI, and *Bcc*I restriction enzymes (New England BioLabs, Ipswich, MA, USA), respectively, at 37 °C for 16 h (Table S2).

### 4.2. Assessment of Plasma PAI-1, Homocysteine, Folate, Total Cholesterol, Uric Acid, and Blood Coagulation Status

Blood samples were collected in anticoagulant containing collection tubes, and the sample tubes were centrifuged for 15 min at 1000× *g* to separate the plasma from whole blood. The PAI-1 levels were examined by the human Serpin E1/PAI-1 immunoassay (R&D Systems, Minneapolis, MN, USA). Homocysteine levels were measured using the IMx fluorescence polarizing immunoassay (Abbott Laboratories, Abbott Park, IL, USA), and folate levels were measured using a radio-assay kit (ACS:180; Bayer, Tarrytown, NY, USA). Total cholesterol and urate levels were determined using commercially available enzymatic colorimetric tests (Roche Diagnostics GmbH, Mannheim, Germany). Platelet counts were measured on a Sysmex XE2100 automated hematology analyzer (Sysmex Corporation, Kobe, Japan). Prothrombin time (PT) and activated partial thromboplastin (aPPT) were measured using an automated photo-optical coagulometer ACL TOP (Mitsubishi Chemical Medience, Tokyo, Japan).

### 4.3. Statistical Analysis

The differences in the genotype and haplotype frequencies between the RPL patients and controls were compared using multivariate logistic regression and Fisher’s exact test, respectively. The OR, AOR, and 95% CIs were used to examine the association between the *MUC4* polymorphisms and RPL risk. Statistical significance was accepted at the *p* < 0.05 level. False-discovery rate (FDR) correction was used to adjust the multiple comparison tests, and associations with an FDR of less than 0.05 were considered as statistically significant. Analyses were performed using Prism 4.0 (GraphPad Software Inc., San Diego, CA, USA) and Medcalc version 12.7.1.0 (Medcalc Software, Mariakerke, Belgium). The haplotype frequencies were estimated with the HAPSTAT program version 3.0. We used StatsDirect (StatsDirect Ltd., Altrincham, United Kingdom) to investigate the correlation of the regression analysis between the *MUC* polymorphisms’ genotype and risk factor.

## Figures and Tables

**Table 1:** The clinical profiles between the RPL patients and control subjects.

Characteristics	Controls (n = 239)	RPL Patients (n = 374)	*p* ^a^
Age (years ± SD)	33.21 ± 5.44	33.21 ± 4.60	0.713 ^b^
BMI (kg/m^2^ ± SD)	21.74 ± 3.47	21.50 ± 3.89	0.587
Previous pregnancy loss (n ± SD)	-	3.31 ± 1.83	N/A
Live birth (n ± SD)	1.76 ± 0.69	-	N/A
Average gestational age (weeks ± SD)	39.29 ± 1.65	7.33 ± 1.85	<0.0001
Homocysteine (μmol/L ± SD)	-	6.97 ± 2.04	N/A
Folate (ng/mL ± SD)	-	14.22 ± 12.00	N/A
Total cholesterol (mg/dL ± SD)	-	187.71 ± 49.89	N/A
Urate (mg/dL ± SD)	-	3.80 ± 0.84	N/A
PAI-1 (ng/mL ± SD)	-	10.52 ± 5.76	N/A
PLT (10^3^/μL ± SD)	237.21 ± 66.69	254.00 ± 58.15	0.009
PT (sec ± SD)	11.54 ± 3.17	11.58 ± 0.86	<0.0001 ^b^
aptt (sec ± SD)	33.44 ± 3.82	32.19 ± 4.19	0.034

Note: RPL, recurrent pregnancy loss; BMI, body mass index; N/A, not applicable; PLT, platelet count; PAI-1, plasminogen activator inhibitor-1, PT, prothrombin time; aPTT, activated partial thromboplastin time. ^a^ Student’s *t* test; ^b^ Mann–Whitney test.

**Table 2:** The genotype frequencies of the *MUC4* gene polymorphisms according to the number of recurrent pregnancy loss.

Genotypes	Controls [n = 239, (%)]	RPL [n = 374, (%)]	AOR (95% CI)	*p*	PL ≥ 3 [n = 138, (%)]	AOR (95% CI)	*p*	PL ≥ 4 [n = 74, (%)]	AOR (95% CI)	*p*
***MUC4* rs882605 G>T**										
GG	171 (71.5)	244 (65.2)	1.000 (reference)		81 (58.7)	1.000 (reference)		43 (58.1)	1.000 (reference)	
GT	63 (26.4)	114 (30.5)	1.269 (0.881–1.827)	0.201	51 (37.0)	1.746 (1.106–2.757)	0.017	29 (39.2)	1.887 (1.081–3.293)	0.026
TT	5 (2.1)	16 (4.3)	2.254 (0.810–6.278)	0.120	6 (4.3)	2.533 (0.751–8.546)	0.134	2 (2.7)	1.589 (0.298–8.475)	0.588
Dominant (GG vs. GT + TT)			1.340 (0.942–1.907)	0.104		1.804 (1.159–2.809)	0.009		1.862 (1.080–3.210)	0.025
Recessive (GG + GT vs. TT)			2.092 (0.756–5.787)	0.155		2.143 (0.641–7.170)	0.216		1.311 (0.249–6.908)	0.750
HWE-*P*	0.774	0.564								
***MUC4* rs1104760 A>G**										
AA	166 (69.5)	234 (62.6)	1.000 (reference)		76 (55.1)	1.000 (reference)		42 (56.8)	1.000 (reference)	
AG	65 (27.2)	127 (34.0)	1.386 (0.968–1.985)	0.075	59 (42.7)	2.001 (1.281–3.125)	0.002	30 (40.5)	1.843 (1.062–3.196)	0.030
GG	8 (3.3)	13 (3.5)	1.125 (0.454–2.785)	0.799	3 (2.2)	0.823 (0.210–3.219)	0.779	2 (2.7)	0.986 (0.201–4.846)	0.986
Dominant (AA vs. AG + GG)			1.361 (0.963–1.924)	0.081		1.880 (1.216–2.905)	0.005		1.757 (1.026–3.008)	0.040
Recessive (AA + AG vs. GG)			1.040 (0.424–2.555)	0.931		0.677 (0.175–2.612)	0.571		0.837 (0.173–4.048)	0.825
HWE-*P*	0.601	0.4								
***MUC4* rs2688513 A>G**										
AA	184 (77.0)	271 (72.5)	1.000 (reference)		99 (71.7)	1.000 (reference)		51 (68.9)	1.000 (reference)	
AG	53 (22.2)	96 (25.7)	1.230 (0.838–1.806)	0.291	35 (25.4)	1.220 (0.745–1.997)	0.430	20 (27.0)	1.351 (0.740–2.467)	0.327
GG	2 (0.8)	7 (1.9)	2.378 (0.488–1.580)	0.283	4 (2.9)	3.815 (0.684–1.271)	0.127	3 (4.1)	5.489 (0.891–3.823)	0.067
Dominant (AA vs. AG + GG)			1.272 (0.872–1.854)	0.212		1.315 (0.815–2.121)	0.261		1.504 (0.844–2.680)	0.167
Recessive (AA + AG vs. GG)			2.261 (0.466–0.982)	0.312		3.687 (0.664–0.473)	0.136		5.172 (0.844–1.691)	0.076
HWE-*P*	0.389	0.654								
***MUC4* rs2258447 C>T**										
CC	165 (69.0)	252 (67.4)	1.000 (reference)		89 (64.5)	1.000 (reference)		49 (66.2)	1.000 (reference)	
CT	63 (26.4)	108 (28.9)	1.121 (0.776–1.619)	0.543	45 (32.6)	1.341 (0.844–2.130)	0.214	24 (32.4)	1.295 (0.733–2.287)	0.374
TT	11 (4.6)	14 (3.7)	0.826 (0.364–1.876)	0.648	4 (2.9)	0.728 (0.220–2.410)	0.603	1 (1.4)	0.323 (0.040–2.607)	0.289
Dominant (CC vs. CT + TT)			1.080 (0.761–1.532)	0.667		1.256 (0.803–1.962)	0.318		1.160 (0.665–2.024)	0.602
Recessive (CC + CT vs. TT)			0.805 (0.358–1.811)	0.600		0.667 (0.205–2.169)	0.500		0.302 (0.038–2.400)	0.257
HWE-*P*	0.128	0.569								
***MUC4* rs2291652 T>C**										
TT	140 (58.6)	203 (54.3)	1.000 (reference)		72 (52.2)	1.000 (reference)		39 (52.7)	1.000 (reference)	
TC	88 (36.8)	147 (39.3)	0.766 (0.358–1.641)	0.493	56 (40.6)	0.703 (0.280–1.765)	0.453	28 (37.8)	0.507 (0.179–1.435)	0.200
CC	11 (4.6)	24 (6.4)	0.665 (0.315–1.401)	0.283	10 (7.2)	0.563 (0.228–1.391)	0.214	7 (9.5)	0.440 (0.160–1.211)	0.112
Dominant (TT vs. TC + CC)			0.704 (0.338–1.464)	0.347		0.618 (0.255–1.495)	0.285		0.465 (0.173–1.248)	0.129
Recessive (TT + TC vs. CC)			0.839 (0.604–1.165)	0.295		0.765 (0.502–1.168)	0.214		0.783 (0.464–1.324)	0.361
HWE-*P*	0.545	0.704								

Note: RPL, recurrent pregnancy loss; AOR, adjusted odds ratio; CI, confidence interval; PL, pregnancy loss; HWE, Hardy–Weinberg equilibrium AOR was adjusted by age.

**Table 3:** The haplotype analysis of the *MUC4* polymorphisms with an increased odds ratio in the RPL patients and control participants.

Haplotypes	Controls (n = 478)	RPL Cases (n = 748)	OR (95% CI)	*p* ^a^	FDR*-P* ^b^
**rs882605 C>A/rs1104760 A>G/rs2688513 A>G/rs2258447 C>T/rs2291652 A>G**					
C-A-A-C-A	286 (59.8)	421 (56.3)	1.000 (reference)		
C-G-A-T-G	2 (0.4)	20 (2.7)	6.793 (1.575–29.300)	0.003	0.023
C-G-G-T-G	5 (1.1)	37 (4.9)	5.027 (1.952–12.950)	0.0001	0.002
A-A-A-C-A	5 (1.0)	61 (8.2)	8.288 (3.289–20.890)	<0.0001	0.002
A-G-G-C-G	0 (0.0)	9 (1.2)	12.910 (0.748–222.900)	0.013	0.078
**rs882605 C>A/rs1104760 A>G/rs2688513 A>G/rs2258447 C>T**					
C-A-A-C	317 (66.4)	475 (63.5)	1.000 (reference)		
C-G-A-T	3 (0.6)	21 (2.8)	4.672 (1.381–15.800)	0.005	0.013
C-G-G-T	5 (1.1)	38 (5.1)	5.072 (1.975–13.030)	0.0002	0.002
A-A-A-C	7 (1.5)	69 (9.2)	6.578 (2.984–14.500)	<0.0001	0.002
A-G-G-C	0 (0.0)	11 (1.4)	15.360 (0.901–261.700)	0.004	0.013
**rs882605 C>A/rs1104760 A>G/rs2688513 A>G/rs2291652 A>G**					
C-A-A-A	291 (60.8)	428 (57.2)	1.000 (reference)		
C-G-G-G	3 (0.6)	39 (5.2)	8.839 (2.705–28.880)	<0.0001	0.001
A-A-A-A	12 (2.6)	63 (8.5)	3.570 (1.891–6.737)	<0.0001	0.001
A-G-G-G	11 (2.3)	33 (4.4)	2.040 (1.014–4.102)	0.042	0.158
**rs882605 C>A/rs1104760 A>G/rs2258447 C>T/rs2291652 A>G**					
C-A-C-A	310 (64.9)	441 (59.0)	1.000 (reference)		
C-G-T-G	7 (1.4)	57 (7.7)	5.724 (2.576–12.720)	<0.0001	0.001
A-A-C-A	7 (1.4)	61 (8.1)	6.126 (2.764–13.570)	<0.0001	0.001
**rs882605 C>A/rs1104760 A>G/rs2688513 A>G**					
C-A-A	331 (69.3)	486 (65.0)	1.000 (reference)		
C-G-G	8 (1.6)	42 (5.6)	3.576 (1.657–7.715)	0.001	0.007
A-A-A	28 (5.8)	77 (10.4)	1.873 (1.189–2.951)	0.006	0.021
A-G-G	11 (2.3)	37 (4.9)	2.291 (1.152–4.556)	0.015	0.026
**rs882605 C>A/rs1104760 A>G/rs2258447 C>T**					
C-A-C	349 (72.9)	496 (66.3)	1.000 (reference)		
C-G-T	8 (1.7)	59 (7.9)	5.189 (2.448–11.000)	<0.0001	0.0004
A-A-C	9 (1.8)	69 (9.3)	5.394 (2.657–10.950)	<0.0001	0.0004
A-G-C	4 (0.8)	17 (2.2)	2.990 (0.997–8.966)	0.040	0.070
**rs882605 C>A/rs1104760 A>G/rs2291652 A>G**					
C-A-A	318 (66.6)	448 (59.9)	1.000 (reference)		
C-G-G	11 (2.3)	58 (7.7)	3.743 (1.933–7.245)	<0.0001	0.0004
A-A-A	16 (3.4)	65 (8.7)	2.884 (1.638–5.077)	0.0001	0.0004
**rs882605 C>A/rs1104760 A>G**					
C-A	366 (76.5)	513 (68.6)	1.000 (reference)		
C-G	39 (8.2)	89 (11.8)	1.628 (1.092–2.428)	0.016	0.024
A-A	31 (6.6)	82 (10.9)	1.887 (1.222–2.914)	0.004	0.012

Note: RPL, recurrent pregnancy loss; OR, odds ratio; CI, confidence interval; FDR, false discovery rate. ^a^ Fisher’s exact test; ^b^ FDR-adjusted *p* value.

**Table 4:** The combination analysis of the *MUC4* polymorphisms between the RPL patients and the control subjects.

Genotypes	Controls (n = 239)	RPL Cases (n = 374)	AOR (95% CI)	*p*
**rs882605 C>A/rs1104760 A>G**				
CC/AA	142 (59.4)	173 (46.3)	1.000 (reference)	
CC/AG	29 (12.1)	66 (17.6)	1.878 (1.150–3.067)	0.012
CA/AA	23 (9.6)	53 (14.2)	1.890 (1.104–3.235)	0.020
CA/AG	32 (13.4)	59 (15.8)	1.501 (0.924–2.438)	0.101
CA/GG	8 (3.3)	2 (0.5)	0.195 (0.041–0.936)	0.041
AA/AA	1 (0.4)	8 (2.1)	6.996 (0.861–6.839)	0.069
AA/AG	4 (1.7)	2 (0.5)	0.417 (0.075–2.316)	0.317
**rs882605 C>A/rs2258447 C>T**				
CC/CC	155 (64.9)	186 (49.7)	1.000 (reference)	
CC/CT	15 (6.3)	52 (13.9)	2.944 (1.592–5.444)	0.001
CC/TT	1 (0.4)	6 (1.6)	4.927 (0.586–1.411)	0.142
CA/CC	9 (3.8)	58 (15.5)	5.441 (2.609–1.350)	<0.0001
CA/CT	47 (19.7)	52 (13.9)	0.918 (0.586–1.439)	0.708
CA/TT	7 (2.9)	4 (1.1)	0.464 (0.132–1.625)	0.230
AA/CC	1 (0.4)	8 (2.1)	6.813 (0.842–5.144)	0.072
AA/CT	1 (0.4)	4 (1.1)	3.439 (0.379–1.190)	0.272
AA/TT	3 (1.3)	4 (1.1)	1.062 (0.233–4.844)	0.938
**rs882605 C>A/rs2291652 A>G**				
CC/AA	125 (52.3)	150 (40.1)	1.000 (reference)	
CC/AG	45 (18.8)	85 (22.7)	1.583 (1.026–2.441)	0.038
CC/GG	1 (0.4)	9 (2.4)	7.676 (0.957–1.583)	0.055
CT/AA	15 (6.3)	45 (12.0)	2.505 (1.333–4.708)	0.004
CT/AG	39 (16.3)	60 (16.0)	1.269 (0.793–2.030)	0.320
CT/GG	9 (3.8)	9 (2.4)	0.849 (0.326–2.208)	0.737
AA/AG	4 (1.7)	2 (0.5)	0.436 (0.078–2.431)	0.344
AA/GG	1 (0.4)	6 (1.6)	4.756 (0.563–0.220)	0.152
**rs1104760 A>G/rs2688513 A>G**				
AA/AA	135 (56.5)	210 (56.1)	1.000 (reference)	
AA/AG	29 (12.1)	24 (6.4)	0.532 (0.297–0.953)	0.034
AG/AA	45 (18.8)	56 (15.0)	0.800 (0.511–1.252)	0.329
AG/AG	20 (8.4)	67 (17.9)	2.157 (1.252–3.719)	0.006
GG/AA	4 (1.7)	5 (1.3)	0.795 (0.208–3.038)	0.738
GG/AG	4 (1.7)	5 (1.3)	0.812 (0.214–3.081)	0.760
**rs1104760 A>G/rs2258447 C>T**				
AA/CC	138 (57.7)	213 (57.0)	1.000 (reference)	
AA/CT	25 (10.5)	21 (5.6)	0.544 (0.293–1.010)	0.054
AG/CC	26 (10.9)	37 (9.9)	0.923 (0.535–1.593)	0.774
AG/CT	33 (13.8)	83 (22.2)	1.627 (1.031–2.570)	0.037
AG/TT	6 (2.5)	7 (1.9)	0.750 (0.247–2.281)	0.613
GG/CC	1 (0.4)	2 (0.5)	1.343 (0.120–4.983)	0.811
GG/CT	5 (2.1)	4 (1.1)	0.507 (0.134–1.924)	0.318
GG/TT	2 (0.8)	7 (1.9)	2.169 (0.440–0.691)	0.342

Note: RPL, recurrent pregnancy loss; AOR, adjusted odds ratio; CI, confidence interval. AOR was adjusted by age.

**Table 5:** Differences in the various clinical parameters according to the *MUC4* polymorphisms in the RPL patients.

Genotypes	PT (sec)	aPTT (sec)	PLT (10^3^/μL)	FSH (mIU/mL)	LH (mIU/mL)
	Mean ± SD	Mean ± SD	Mean ± SD	Mean ± SD	Mean ± SD
***MUC4*** **rs882605 C>A**					
CC	11.91 ± 3.68	33.82 ± 3.81	234.03 ± 67.57	8.39 ± 3.04	3.22 ± 1.60
CA	10.72 ± 1.15	32.94 ± 3.85	245.64 ± 66.70	7.20 ± 2.09	3.56 ± 2.10
AA	10.50 ± 0.71	29.48 ± 0.74	246.25 ± 26.41	9.45 ± 1.34	4.60 ± 3.25
***p* ^a^**	0.384	0.240	0.582	0.138	0.406
***MUC4*** **rs1104760 A>G**					
AA	12.22 ± 3.95	34.21 ± 4.02	234.89 ± 67.22	8.23 ± 3.02	3.23 ± 1.65
AG	10.51 ± 0.92	32.43 ± 3.32	245.37 ± 65.85	7.74 ± 2.22	3.67 ± 2.20
GG	12.30 ± 0.00	30.50 ± 0.00	202.67 ± 57.83	7.40 ± 0.71	4.10 ± 0.71
***p* ^a^**	0.013 ^b^	0.148	0.431	0.724	0.499
***MUC4*** **rs2688513 A>G**					
AA	11.66 ± 3.53	33.93 ± 4.04	233.67 ± 70.14	8.24 ± 3.04	3.33 ± 1.58
AG	11.07 ± 1.01	31.59 ± 1.97	246.92 ± 48.42	7.70 ± 2.16	3.45 ± 2.23
GG	N/A	N/A	313.50 ± 61.52	8.35 ± 2.47	1.35 ± 0.50
***p* ^a^**	0.549	0.036 ^b^	0.151	0.710	0.262
***MUC4*** **rs2258447 C>T**					
CC	11.71 ± 3.31	34.19 ± 3.81	232.99 ± 64.30	8.39 ± 3.08	3.22 ± 1.61
CT	11.25 ± 3.26	32.45 ± 3.66	247.80 ± 75.77	7.27 ± 2.01	3.58 ± 2.10
TT	10.94 ± 1.00	29.86 ± 0.65	244.50 ± 46.15	8.20 ± 1.83	3.85 ± 2.26
***p* ^a^**	0.820	0.041	0.421	0.222	0.551
***MUC4*** **rs2291652 A>G**					
AA	11.18 ± 1.04	32.93 ± 4.65	222.86 ± 56.47	8.03 ± 1.98	3.63 ± 2.69
AG	11.15 ± 3.97	32.15 ± 2.99	247.65 ± 68.56	8.38 ± 1.91	3.62 ± 1.98
GG	11.83 ± 2.70	34.35 ± 4.08	232.28 ± 66.02	8.00 ± 3.24	3.17 ± 1.57
***p* ^a^**	0.716	0.088	0.295	0.819	0.449

Note: PT, prothrombin time; aPTT, activated partial thromboplastin time; PLT, platelet count; FSH, follicle stimulating hormone; LH, luteinizing hormone; N/A, not applicable. ^a^ Calculated using ANOVA. ^b^ Calculated using the Kruskal–Wallis test.

## Data Availability

Data sharing not applicable.

## Supplementary Materials

The following supporting information can be downloaded at: https://www.mdpi.com/article/10.3390/genes13060937/s1, Figure S1: Schematic of *MUC4* polymorphism locations; Table S1: Haplotype analysis of *MUC4* polymorphisms in RPL patients and control participants; Table S2: The PCR primer sequence and enzyme used for SNP genotyping.
