# Supplementary material for: Association Study between Mucin 4 (MUC4) Polymorphisms and Idiopathic Recurrent Pregnancy Loss in a Korean Population
Source: Genes (Basel). 2022 May 24;13(6):937. doi: 10.3390/genes13060937 (PMC9222798; doi:10.3390/genes13060937)
Supplement: Supplementary file 1 [file genes-13-00937-s001.zip › genes-1707196-supplementary.pdf]

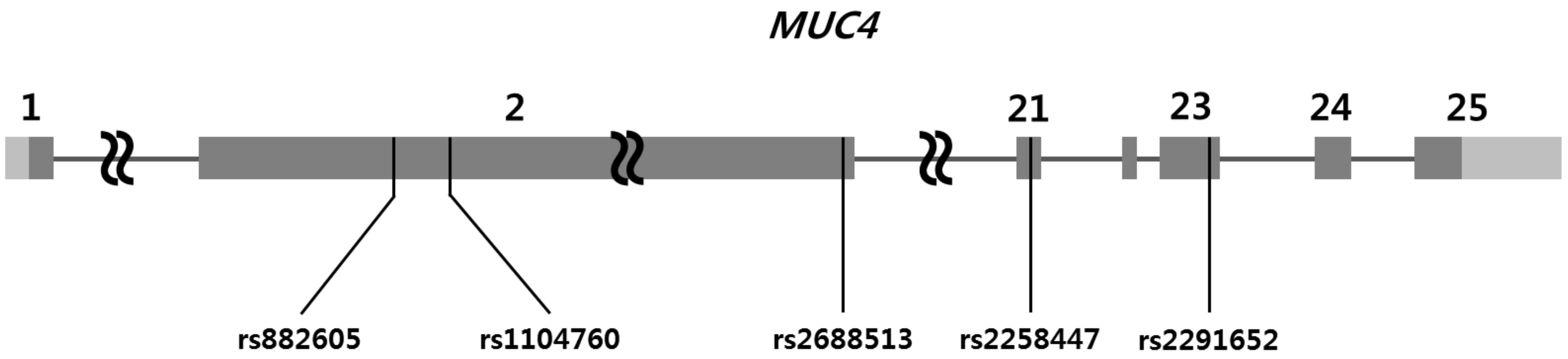

Supplementary Figure S1. Schematic of *MUC4* polymorphism locations.

Supplementary Table S1. Haplotype analysis of *MUC4* polymorphisms in RPL patients and control participants

Haplotypes	Controls (n= 478)	RPL cases (n=748)	OR (95% CI)	P^a	FDR-P^b
**rs882605 C>A / rs1104760 A>G / rs2688513 A>G / rs2258447 C>T / rs2291652 A>G**					
C-A-A-C-A	286 (59.8)	421 (56.3)	1.000 (reference)		
C-A-A-C-G	34 (7.1)	55 (7.4)	1.099 (0.698 - 1.729)	0.683	0.829
C-A-A-T-A	3 (0.7)	6 (0.8)	1.359 (0.337 - 5.479)	0.746	0.861
C-A-A-T-G	9 (2.0)	5 (0.6)	0.377 (0.125 - 1.138)	0.073	0.234
C-A-G-C-A	25 (5.3)	20 (2.6)	0.544 (0.296 - 0.997)	0.046	0.197
C-A-G-C-G	4 (0.8)	1 (0.2)	0.170 (0.019 - 1.528)	0.164	0.354
C-A-G-T-A	4 (0.9)	2 (0.3)	0.340 (0.062 - 1.868)	0.231	0.462
C-A-G-T-G	0 (0.0)	5 (0.6)	7.477 (0.412 - 135.800)	0.086	0.235
C-G-A-C-A	22 (4.5)	24 (3.2)	0.741 (0.408 - 1.347)	0.355	0.666
C-G-A-C-G	6 (1.2)	1 (0.1)	0.113 (0.014 - 0.946)	0.021	0.105
C-G-A-T-A	1 (0.3)	1 (0.2)	0.679 (0.042 - 10.910)	1.000	1.000
C-G-A-T-G	2 (0.4)	20 (2.7)	6.793 (1.575 - 29.300)	0.003	0.023
C-G-G-C-A	4 (0.8)	3 (0.4)	0.510 (0.113 - 2.294)	0.451	0.752
C-G-G-C-G	0 (0.0)	1 (0.2)	2.039 (0.083 - 50.270)	1.000	1.000
C-G-G-T-G	5 (1.1)	37 (4.9)	5.027 (1.952 - 12.950)	0.0001	0.002
A-A-A-C-A	5 (1.0)	61 (8.2)	8.288 (3.289 - 20.890)	<0.0001	0.002
A-A-A-C-G	2 (0.5)	6 (0.8)	2.038 (0.408 - 10.170)	0.485	0.766
A-A-A-T-A	9 (1.8)	1 (0.2)	0.075 (0.010 - 0.599)	0.002	0.020
A-A-A-T-G	11 (2.3)	7 (0.9)	0.432 (0.166 - 1.129)	0.078	0.234
A-A-G-C-A	2 (0.3)	0 (0.0)	0.136 (0.006 - 2.844)	0.165	0.354
A-A-G-C-G	0 (0.0)	1 (0.1)	2.039 (0.083 - 50.270)	1.000	1.000
A-A-G-T-A	1 (0.3)	3 (0.4)	2.038 (0.211 - 19.700)	0.651	0.829
A-A-G-T-G	2 (0.4)	1 (0.1)	0.340 (0.031 - 3.766)	0.569	0.813
A-G-A-C-A	1 (0.2)	4 (0.5)	2.717 (0.302 - 24.450)	0.653	0.829
A-G-A-C-G	3 (0.7)	3 (0.5)	0.679 (0.136 - 3.391)	0.691	0.829
A-G-A-T-A	4 (0.9)	3 (0.4)	0.510 (0.113 - 2.294)	0.451	0.752
A-G-A-T-G	23 (4.8)	19 (2.6)	0.561 (0.300 - 1.050)	0.067	0.234
A-G-G-C-A	0 (0.0)	2 (0.3)	3.399 (0.162 - 71.110)	0.518	0.777
A-G-G-C-G	0 (0.0)	9 (1.2)	12.910 (0.748 - 222.900)	0.013	0.078
A-G-G-T-A	1 (0.2)	2 (0.3)	1.359 (0.123 - 15.060)	1.000	1.000
A-G-G-T-G	9 (1.9)	24 (3.3)	1.812 (0.830 - 3.955)	0.148	0.354
**rs882605 C>A / rs1104760 A>G / rs2688513 A>G / rs2258447 C>T**					
C-A-A-C	317 (66.4)	475 (63.5)	1.000 (reference)		
C-A-A-T	12 (2.6)	10 (1.3)	0.556 (0.237 - 1.303)	0.171	0.263
C-A-G-C	31 (6.5)	21 (2.8)	0.452 (0.255 - 0.801)	0.005	0.013
C-A-G-T	4 (0.9)	8 (1.0)	1.335 (0.398 - 4.471)	0.771	0.964
C-G-A-C	28 (5.9)	26 (3.5)	0.620 (0.357 - 1.077)	0.087	0.163

C-G-A-T	3 (0.6)	21 (2.8)	4.672 (1.381 - 15.800)	0.005	0.013
C-G-G-C	4 (0.7)	4 (0.5)	0.667 (0.166 - 2.689)	0.720	0.964
C-G-G-T	5 (1.1)	38 (5.1)	5.072 (1.975 - 13.030)	0.0002	0.002
A-A-A-C	7 (1.5)	69 (9.2)	6.578 (2.984 - 14.500)	<0.0001	0.002
A-A-A-T	22 (4.6)	9 (1.2)	0.273 (0.124 - 0.601)	0.001	0.005
A-A-G-C	1 (0.3)	1 (0.1)	0.667 (0.042 - 10.720)	1.000	1.000
A-A-G-T	1 (0.3)	3 (0.4)	2.002 (0.207 - 19.340)	1.000	1.000
A-G-A-C	4 (0.9)	6 (0.8)	1.001 (0.280 - 3.577)	1.000	1.000
A-G-A-T	27 (5.7)	22 (3.0)	0.544 (0.304 - 0.972)	0.037	0.079
A-G-G-C	0 (0.0)	11 (1.4)	15.360 (0.901 - 261.700)	0.004	0.013
A-G-G-T	10 (2.1)	25 (3.4)	1.668 (0.790 - 3.522)	0.175	0.263
**rs882605 C>A / rs1104760 A>G / rs2688513 A>G / rs2291652 A>G**					
C-A-A-A	291 (60.8)	428 (57.2)	1.000 (reference)		
C-A-A-G	40 (8.3)	59 (7.8)	1.003 (0.654 - 1.539)	0.990	1.000
C-A-G-A	28 (5.9)	20 (2.7)	0.486 (0.268 - 0.879)	0.015	0.075
C-A-G-G	7 (1.5)	8 (1.0)	0.777 (0.279 - 2.167)	0.629	0.726
C-G-A-A	23 (4.9)	28 (3.7)	0.828 (0.467 - 1.466)	0.516	0.678
C-G-A-G	8 (1.8)	19 (2.5)	1.615 (0.697 - 3.739)	0.259	0.470
C-G-G-A	5 (1.0)	3 (0.3)	0.408 (0.097 - 1.721)	0.282	0.470
C-G-G-G	3 (0.6)	39 (5.2)	8.839 (2.705 - 28.880)	<0.0001	0.001
A-A-A-A	12 (2.6)	63 (8.5)	3.570 (1.891 - 6.737)	<0.0001	0.001
A-A-A-G	16 (3.3)	14 (1.9)	0.595 (0.286 - 1.238)	0.161	0.345
A-A-G-A	3 (0.7)	2 (0.3)	0.453 (0.075 - 2.731)	0.401	0.602
A-A-G-G	0 (0.0)	1 (0.2)	2.041 (0.083 - 50.310)	1.000	1.000
A-G-A-A	5 (1.1)	5 (0.7)	0.680 (0.195 - 2.370)	0.542	0.678
A-G-A-G	25 (5.2)	23 (3.1)	0.626 (0.348 - 1.124)	0.114	0.342
A-G-G-A	0 (0.0)	4 (0.6)	6.123 (0.328 - 114.200)	0.153	0.345
A-G-G-G	11 (2.3)	33 (4.4)	2.040 (1.014 - 4.102)	0.042	0.158
**rs882605 C>A / rs1104760 A>G / rs2258447 C>T / rs2291652 A>G**					
C-A-C-A	310 (64.9)	441 (59.0)	1.000 (reference)		
C-A-C-G	38 (8.0)	56 (7.5)	1.036 (0.669 - 1.604)	0.874	0.874
C-A-T-A	8 (1.6)	8 (1.1)	0.703 (0.261 - 1.894)	0.484	0.660
C-A-T-G	9 (1.8)	9 (1.3)	0.703 (0.276 - 1.791)	0.477	0.660
C-G-C-A	26 (5.5)	27 (3.6)	0.730 (0.418 - 1.275)	0.313	0.535
C-G-C-G	5 (1.1)	2 (0.3)	0.281 (0.054 - 1.459)	0.134	0.335
C-G-T-A	2 (0.3)	1 (0.2)	0.352 (0.032 - 3.896)	0.573	0.716
C-G-T-G	7 (1.4)	57 (7.7)	5.724 (2.576 - 12.720)	<0.0001	0.001
A-A-C-A	7 (1.4)	61 (8.1)	6.126 (2.764 - 13.570)	<0.0001	0.001
A-A-C-G	2 (0.5)	7 (1.0)	2.460 (0.508 - 11.930)	0.321	0.535
A-A-T-A	10 (2.0)	4 (0.5)	0.281 (0.087 - 0.905)	0.029	0.145
A-A-T-G	13 (2.8)	8 (1.1)	0.433 (0.177 - 1.056)	0.073	0.274

A-G-C-A	1 (0.2)	6 (0.7)	4.218 (0.505 - 35.230)	0.250	0.535
A-G-C-G	3 (0.7)	12 (1.6)	2.812 (0.787 - 10.050)	0.116	0.335
A-G-T-A	5 (1.1)	5 (0.7)	0.703 (0.202 - 2.450)	0.749	0.864
A-G-T-G	32 (6.8)	43 (5.7)	0.945 (0.584 - 1.527)	0.807	0.865
**rs882605 C>A / rs2688513 A>G / rs2258447 C>T / rs2291652 A>G**					
C-A-C-A	305 (63.7)	445 (59.4)	1.000 (reference)		
C-A-C-G	41 (8.6)	57 (7.6)	0.953 (0.622 - 1.461)	0.825	0.873
C-A-T-A	5 (1.1)	8 (1.1)	1.097 (0.355 - 3.385)	0.873	0.873
C-A-T-G	11 (2.2)	24 (3.2)	1.495 (0.722 - 3.099)	0.276	0.460
C-G-C-A	32 (6.6)	21 (2.8)	0.450 (0.255 - 0.795)	0.005	0.023
C-G-C-G	3 (0.6)	3 (0.4)	0.685 (0.137 - 3.420)	0.692	0.818
C-G-T-A	5 (1.0)	2 (0.3)	0.274 (0.053 - 1.423)	0.129	0.276
C-G-T-G	4 (0.9)	43 (5.8)	7.368 (2.617 - 20.740)	<0.0001	0.001
A-A-C-A	6 (1.3)	65 (8.7)	7.425 (3.177 - 17.360)	<0.0001	0.001
A-A-C-G	5 (1.1)	9 (1.2)	1.234 (0.409 - 3.718)	0.709	0.818
A-A-T-A	12 (2.5)	4 (0.6)	0.229 (0.073 - 0.715)	0.009	0.023
A-A-T-G	36 (7.6)	26 (3.5)	0.495 (0.293 - 0.837)	0.008	0.023
A-G-C-A	1 (0.3)	4 (0.5)	2.742 (0.305 - 24.660)	0.653	0.818
A-G-C-G	0 (0.0)	10 (1.3)	14.400 (0.840 - 246.800)	0.007	0.023
A-G-T-A	2 (0.5)	5 (0.6)	1.713 (0.330 - 8.893)	0.707	0.818
A-G-T-G	10 (2.0)	24 (3.2)	1.645 (0.775 - 3.490)	0.190	0.356
**rs1104760 A>G / rs2688513 A>G / rs2258447 C>T / rs2291652 A>G**					
A-A-C-A	290 (60.8)	482 (64.4)	1.000 (reference)		
A-A-C-G	36 (7.6)	62 (8.3)	1.036 (0.670 - 1.602)	0.873	1.000
A-A-T-A	12 (2.5)	7 (1.0)	0.351 (0.137 - 0.902)	0.023	0.058
A-A-T-G	21 (4.3)	12 (1.6)	0.344 (0.167 - 0.709)	0.003	0.020
A-G-C-A	27 (5.7)	19 (2.5)	0.423 (0.231 - 0.775)	0.004	0.020
A-G-C-G	4 (0.8)	3 (0.4)	0.451 (0.100 - 2.031)	0.435	0.653
A-G-T-A	5 (1.1)	5 (0.7)	0.602 (0.173 - 2.097)	0.420	0.653
A-G-T-G	2 (0.4)	6 (0.8)	1.805 (0.362 - 9.006)	0.717	0.919
G-A-C-A	23 (4.8)	29 (3.9)	0.759 (0.431 - 1.337)	0.338	0.634
G-A-C-G	9 (1.9)	3 (0.3)	0.201 (0.054 - 0.747)	0.013	0.039
G-A-T-A	6 (1.3)	4 (0.6)	0.401 (0.112 - 1.434)	0.191	0.409
G-A-T-G	24 (5.1)	40 (5.3)	1.003 (0.592 - 1.698)	0.992	1.000
G-G-C-A	4 (0.8)	5 (0.7)	0.752 (0.200 - 2.824)	0.735	0.919
G-G-C-G	0 (0.0)	11 (1.4)	13.850 (0.812 - 236.000)	0.009	0.034
G-G-T-A	1 (0.2)	2 (0.3)	1.203 (0.109 - 13.340)	1.000	1.000
G-G-T-G	15 (3.1)	60 (8.0)	2.407 (1.342 - 4.317)	0.003	0.020
**rs882605 C>A / rs1104760 A>G / rs2688513 A>G**					
C-A-A	331 (69.3)	486 (65.0)			
C-A-G	35 (7.2)	27 (3.7)	0.525 (0.312 - 0.885)	0.014	0.026

C-G-A	32 (6.6)	46 (6.2)	0.979 (0.610 - 1.570)	0.930	0.930
C-G-G	8 (1.6)	42 (5.6)	3.576 (1.657 - 7.715)	0.001	0.007
A-A-A	28 (5.8)	77 (10.4)	1.873 (1.189 - 2.951)	0.006	0.021
A-A-G	4 (0.8)	4 (0.5)	0.681 (0.169 - 2.743)	0.587	0.685
A-G-A	30 (6.4)	28 (3.8)	0.636 (0.373 - 1.084)	0.094	0.132
A-G-G	11 (2.3)	37 (4.9)	2.291 (1.152 - 4.556)	0.015	0.026
**rs882605 C>A / rs1104760 A>G / rs2258447 C>T**					
C-A-C	349 (72.9)	496 (66.3)			
C-A-T	17 (3.5)	17 (2.3)	0.704 (0.354 - 1.398)	0.313	0.365
C-G-C	32 (6.6)	30 (4.0)	0.660 (0.394 - 1.106)	0.112	0.157
C-G-T	8 (1.7)	59 (7.9)	5.189 (2.448 - 11.000)	<0.0001	0.000
A-A-C	9 (1.8)	69 (9.3)	5.394 (2.657 - 10.950)	<0.0001	0.000
A-A-T	23 (4.8)	12 (1.7)	0.367 (0.180 - 0.748)	0.004	0.009
A-G-C	4 (0.8)	17 (2.2)	2.990 (0.997 - 8.966)	0.040	0.070
A-G-T	37 (7.8)	47 (6.4)	0.894 (0.569 - 1.405)	0.626	0.626
**rs882605 C>A / rs1104760 A>G / rs2291652 A>G**					
C-A-A	318 (66.6)	448 (59.9)			
C-A-G	47 (9.9)	66 (8.8)	0.997 (0.668 - 1.488)	0.987	0.987
C-G-A	28 (5.9)	30 (4.0)	0.761 (0.446 - 1.298)	0.314	0.621
C-G-G	11 (2.3)	58 (7.7)	3.743 (1.933 - 7.245)	<0.0001	0.000
A-A-A	16 (3.4)	65 (8.7)	2.884 (1.638 - 5.077)	0.0001	0.0004
A-A-G	15 (3.2)	15 (2.1)	0.710 (0.342 - 1.473)	0.355	0.621
A-G-A	5 (1.1)	10 (1.3)	1.420 (0.481 - 4.194)	0.524	0.734
A-G-G	36 (7.6)	56 (7.4)	1.104 (0.709 - 1.719)	0.661	0.771
**rs882605 C>A / rs2688513 A>G / rs2258447 C>T**					
C-A-C	345 (72.2)	500 (66.9)			
C-A-T	16 (3.3)	32 (4.2)	1.380 (0.746 - 2.554)	0.303	0.303
C-G-C	35 (7.4)	24 (3.2)	0.473 (0.276 - 0.810)	0.005	0.009
C-G-T	9 (1.9)	46 (6.2)	3.527 (1.704 - 7.301)	0.0003	0.001
A-A-C	12 (2.5)	75 (10.0)	4.313 (2.309 - 8.055)	<0.0001	0.001
A-A-T	49 (10.2)	31 (4.2)	0.437 (0.273 - 0.699)	0.0004	0.001
A-G-C	1 (0.2)	13 (1.8)	8.970 (1.167 - 68.920)	0.011	0.015
A-G-T	12 (2.5)	27 (3.6)	1.553 (0.776 - 3.107)	0.211	0.246
**rs882605 C>A / rs2688513 A>G / rs2291652 A>G**					
C-A-A	312 (65.3)	455 (60.8)			
C-A-G	51 (10.7)	77 (10.3)	1.035 (0.707 - 1.517)	0.859	0.859
C-G-A	35 (7.2)	22 (2.9)	0.431 (0.248 - 0.749)	0.002	0.005
C-G-G	7 (1.5)	48 (6.4)	4.702 (2.100 - 10.530)	<0.0001	0.001
A-A-A	18 (3.7)	68 (9.2)	2.590 (1.511 - 4.442)	0.0004	0.001
A-A-G	40 (8.4)	37 (5.0)	0.634 (0.397 - 1.015)	0.056	0.098
A-G-A	4 (0.8)	8 (1.1)	1.371 (0.409 - 4.595)	0.607	0.708

A-G-G	12 (2.4)	32 (4.3)	1.829 (0.927 - 3.606)	0.078	0.109
**rs882605 C>A / rs2258447 C>T / rs2291652 A>G**					
C-C-A	336 (70.3)	466 (62.3)			
C-C-G	44 (9.3)	59 (7.8)	0.967 (0.639 - 1.464)	0.873	0.873
C-T-A	10 (2.0)	9 (1.3)	0.649 (0.261 - 1.615)	0.349	0.407
C-T-G	15 (3.2)	68 (9.1)	3.269 (1.836 - 5.818)	<0.0001	0.000
A-C-A	8 (1.6)	68 (9.2)	6.129 (2.906 - 12.920)	<0.0001	0.000
A-C-G	5 (1.0)	19 (2.6)	2.740 (1.013 - 7.413)	0.039	0.077
A-T-A	15 (3.1)	9 (1.3)	0.433 (0.187 - 1.001)	0.044	0.077
A-T-G	46 (9.6)	49 (6.6)	0.768 (0.502 - 1.176)	0.224	0.314
**rs1104760 A>G / rs2688513 A>G / rs2258447 C>T**					
A-A-C	325 (68.0)	544 (72.7)			
A-A-T	33 (7.0)	19 (2.6)	0.344 (0.192 - 0.615)	0.0002	0.001
A-G-C	32 (6.8)	21 (2.9)	0.392 (0.222 - 0.692)	0.001	0.004
A-G-T	6 (1.3)	11 (1.4)	1.095 (0.401 - 2.990)	0.859	0.859
G-A-C	33 (6.9)	32 (4.2)	0.579 (0.350 - 0.960)	0.033	0.058
G-A-T	30 (6.2)	43 (5.8)	0.856 (0.527 - 1.392)	0.531	0.620
G-G-C	3 (0.6)	15 (2.0)	2.987 (0.858 - 10.400)	0.071	0.099
G-G-T	16 (3.3)	63 (8.4)	2.352 (1.336 - 4.142)	0.002	0.005
**rs1104760 A>G / rs2688513 A>G / rs2291652 A>G**					
A-A-A	302 (63.2)	491 (65.7)			
A-A-G	57 (11.9)	73 (9.7)	0.788 (0.541 - 1.146)	0.212	0.357
A-G-A	33 (6.8)	23 (3.0)	0.429 (0.247 - 0.744)	0.002	0.007
A-G-G	6 (1.2)	9 (1.2)	0.923 (0.325 - 2.618)	0.880	0.880
G-A-A	28 (6.0)	32 (4.3)	0.703 (0.415 - 1.191)	0.188	0.357
G-A-G	34 (7.1)	42 (5.6)	0.760 (0.473 - 1.221)	0.255	0.357
G-G-A	5 (1.1)	7 (0.9)	0.861 (0.271 - 2.738)	0.800	0.880
G-G-G	14 (2.8)	72 (9.6)	3.163 (1.753 - 5.708)	<0.0001	0.001
**rs1104760 A>G / rs2258447 C>T / rs2291652 A>G**					
A-C-A	317 (66.3)	501 (67.0)			
A-C-G	41 (8.5)	64 (8.6)	0.988 (0.651 - 1.498)	0.954	0.971
A-T-A	17 (3.7)	12 (1.6)	0.447 (0.211 - 0.948)	0.031	0.093
A-T-G	22 (4.6)	18 (2.4)	0.518 (0.273 - 0.981)	0.040	0.093
G-C-A	27 (5.6)	33 (4.4)	0.773 (0.456 - 1.311)	0.339	0.549
G-C-G	9 (1.8)	14 (1.8)	0.984 (0.421 - 2.301)	0.971	0.971
G-T-A	7 (1.4)	7 (0.9)	0.633 (0.220 - 1.821)	0.392	0.549
G-T-G	39 (8.2)	99 (13.3)	1.606 (1.080 - 2.388)	0.018	0.093
**rs2688513 A>G / rs2258447 C>T / rs2291652 A>G**					
A-C-A	310 (64.9)	510 (68.2)			
A-C-G	47 (9.9)	65 (8.6)	0.841 (0.563 - 1.255)	0.396	0.396
A-T-A	18 (3.7)	12 (1.6)	0.405 (0.193 - 0.853)	0.014	0.033

A-T-G	46 (9.6)	51 (6.9)	0.674 (0.442 - 1.029)	0.066	0.092
G-C-A	34 (7.0)	24 (3.2)	0.429 (0.250 - 0.737)	0.002	0.007
G-C-G	2 (0.4)	13 (1.8)	3.951 (0.885 - 17.630)	0.052	0.091
G-T-A	7 (1.4)	7 (0.9)	0.608 (0.211 - 1.750)	0.351	0.396
G-T-G	15 (3.1)	66 (8.8)	2.675 (1.500 - 4.768)	0.001	0.007
**rs882605 C>A / rs1104760 A>G**					
C-A	366 (76.5)	513 (68.6)			
C-G	39 (8.2)	89 (11.8)	1.628 (1.092 - 2.428)	0.016	0.024
A-A	31 (6.6)	82 (10.9)	1.887 (1.222 - 2.914)	0.004	0.012
A-G	42 (8.7)	64 (8.6)	1.087 (0.720 - 1.641)	0.691	0.691
**rs882605 C>A / rs2688513 A>G**					
C-A	363 (76.0)	532 (71.1)			
C-G	42 (8.7)	70 (9.4)	1.137 (0.758 - 1.705)	0.534	0.534
A-A	58 (12.1)	106 (14.2)	1.247 (0.882 - 1.764)	0.212	0.318
A-G	15 (3.2)	40 (5.3)	1.820 (0.990 - 3.343)	0.051	0.153
**rs882605 C>A / rs2258447 C>T**					
C-C	380 (79.6)	525 (70.1)			
C-T	25 (5.2)	77 (10.3)	2.229 (1.393 - 3.568)	0.001	0.002
A-C	13 (2.6)	87 (11.7)	4.844 (2.665 - 8.806)	<0.0001	0.000
A-T	60 (12.6)	59 (7.8)	0.712 (0.485 - 1.044)	0.081	0.081
**rs882605 C>A / rs2291652 A>G**					
C-A	346 (72.5)	477 (63.7)			
C-G	59 (12.3)	125 (16.8)	1.537 (1.095 - 2.157)	0.013	0.020
A-A	22 (4.5)	76 (10.2)	2.506 (1.528 - 4.108)	0.0002	0.001
A-G	51 (10.8)	69 (9.3)	0.981 (0.666 - 1.446)	0.924	0.924
**rs1104760 A>G / rs2688513 A>G**					
A-A	359 (75.1)	564 (75.4)			
A-G	38 (8.0)	31 (4.2)	0.519 (0.317 - 0.850)	0.008	0.012
G-A	62 (13.0)	74 (9.9)	0.760 (0.529 - 1.092)	0.137	0.137
G-G	19 (3.9)	79 (10.5)	2.647 (1.576 - 4.443)	0.0001	0.0003
**rs1104760 A>G / rs2258447 C>T**					
A-C	357 (74.8)	565 (75.6)			
A-T	40 (8.3)	30 (4.0)	0.474 (0.290 - 0.775)	0.002	0.006
G-C	36 (7.5)	47 (6.3)	0.825 (0.524 - 1.299)	0.405	0.405
G-T	45 (9.5)	106 (14.2)	1.488 (1.025 - 2.162)	0.036	0.054
**rs1104760 A>G / rs2291652 A>G**					
A-A	334 (70.0)	513 (68.6)			
A-G	63 (13.1)	82 (10.9)	0.847 (0.594 - 1.210)	0.362	0.362
G-A	34 (7.0)	40 (5.3)	0.766 (0.475 - 1.235)	0.273	0.362
G-G	47 (9.9)	113 (15.2)	1.565 (1.084 - 2.260)	0.016	0.048
**rs2688513 A>G / rs2258447 C>T**					

A-C	357 (74.8)	575 (76.9)			
A-T	64 (13.3)	63 (8.4)	0.611 (0.421 - 0.887)	0.009	0.014
G-C	36 (7.4)	37 (5.0)	0.638 (0.396 - 1.029)	0.063	0.063
G-T	21 (4.5)	73 (9.7)	2.158 (1.305 - 3.569)	0.002	0.006
**rs2688513 A>G / rs2291652 A>G**					
A-A	330 (69.0)	523 (69.9)			
A-G	91 (19.1)	115 (15.4)	0.797 (0.586 - 1.085)	0.149	0.149
G-A	38 (8.0)	30 (4.0)	0.498 (0.303 - 0.820)	0.005	0.008
G-G	19 (3.9)	80 (10.7)	2.657 (1.581 - 4.464)	0.0001	0.0003
**rs2258447 C>T / rs2291652 A>G**					
C-A	344 (71.9)	534 (71.4)			
C-G	49 (10.3)	78 (10.4)	1.025 (0.700 - 1.503)	0.897	0.897
T-A	24 (5.1)	19 (2.5)	0.510 (0.275 - 0.945)	0.030	0.089
T-G	61 (12.7)	117 (15.7)	1.236 (0.881 - 1.732)	0.219	0.329

Note: RPL, recurrent pregnancy loss; OR, odds ratio; CI, confidence interval; FDR, false discovery rate.

[a] Fisher's exact test; [b] FDR-adjusted *P* value.

Supplementary Table S2. The PCR primer sequence and enzyme used for SNP genotyping

SNP	Primer	Annealing temperature	Enzyme	Reaction temperature
MUC4 rs882605 C>A	Forward: 5′ – CAC CTC AGC AGC CTT AGT AAT A – 3′<br>Reverse: 5′ – TGA TGT TGT AAC CGG TGT G – 3′	58°C	*Ssp* I	37°C
MUC4 rs1104760 A>G	Forward: 5′ – CCA CTT ACA GAT AGT GAT GTC TCC – 3′<br>Reverse: 5′ – CTC AAA TCA ACA CCC TCA ACA C – 3′	55°C	*Bcc* I	
MUC4 rs2688513 A>G	Forward: 5' - ACA TAA AGG CGA GGC AGT TG – 3'<br>Reverse: 5' - ACC CCT CTT CCT GTC ACC A – 3'	54°C	*Bst* NI	
MUC4 rs2291652 A>G	Forward: 5' - GAA GCC CCA TCC AAC ACT GG – 3'<br>Reverse: 5' - GAC TCA CGG GCT GTC ACA TC – 3'	55°C	*Bcc* I	
MUC4 rs2258447 C>T	TaqMan probe SNP genotyping assay kit			
